# Vascular Anomaly Presenting as Neurological Crisis: A Case Report of Dolichoectasia-Induced Seizures

**DOI:** 10.7759/cureus.68443

**Published:** 2024-09-02

**Authors:** Manjeet Kothari, Shilpa A Gaidhane, Vinit Deolikar, Rushikesh H Dhondge, Rajvardhan Patil

**Affiliations:** 1 Department of Medicine, Datta Meghe Institute of Higher Education and Research, Wardha, IND; 2 School of Epidemiology and Public Health, Jawaharlal Nehru Medical College, Datta Meghe Institute of Higher Education and Research, Wardha, IND

**Keywords:** internal carotid artery (ica), mri, gtcs, seizures, intracranial arterial dolichoectasia

## Abstract

A rare brain vasculature illness called dolichoectasia (DE) causes the blood vessels to enlarge and elongate. DE describes an irregular enlargement and dilatation of arteries; this condition primarily affects big blood vessels such as the internal carotid and vertebrobasilar arteries. A range of neurological symptoms may result from this illness because of the compression of nearby structures or changed hemodynamics. Although it is documented more commonly in the vertebrobasilar circulation, rarely it can be seen in the anterior circulation, as in our case.

The brainstem structures and cranial nerves are compressed in DE, resulting in turbulent blood flow that causes vertigo and tinnitus and frequently damages the brain's small blood vessels. One risk factor for ischemic stroke is DE. The occurrence of seizures in DE is not so common. Here, we report a case of a 20-year-old female, bioengineering UG with generalized tonic-clonic seizure diagnosed to have DE on MRI.

## Introduction

Irregular enlargement and dilation of arteries is broadly referred to as dolichoectasia (DE). The big blood vessels involved in cerebral circulation such as the internal carotid and vertebrobasilar arteries are commonly affected. A range of neurological symptoms may result from this illness because of the compression of nearby structures or changed hemodynamics.

The prevalence of DE has been reported to be 0.05%-0.06%, with preferential involvement of the vertebrobasilar circulation [1] in contrast with the relatively rare involvement of the anterior circulation [2]. Although a clear etiology for DE has not been established, possible mechanisms may include hypertension-induced atherosclerosis, congenital factors, or infections; however, none of these have been proven as definitive causes. The most prevalent age group associated with DE is middle to old age, typically between 50 and 70 years. However, this condition is very rarely seen in younger individuals, and when it does occur in young patients, it is most often related to a developmental abnormality.

Patients with DE can present with a range of unusual clinical symptoms. Patients exhibiting symptoms may include those with an ischemic stroke, brainstem compression, cranial nerve complaints, hydrocephalus and hemorrhage, or obstructive hydrocephalus. Imaging tests are the primary means of diagnosing DE. According to a study, ischemic stroke is the most frequent clinical sign of DE and the main reason why DE patients die. Posterior circulation infarction is the predominant ischemic stroke subtype in people with DE, and DE is a separate risk factor for the condition.

A seizure characterized by a tonic phase and clonic muscle contractions is called a generalized tonic-clonic seizure (GTCS) [3].

The occurrence of GTCS is not a common feature of DE; hence, we report a case of a 20-year-old female who presented with generalized movements of all four limbs and was diagnosed with DE.

## Case presentation

A 20-year-old female was brought to our hospital emergency department with an ongoing episode of tonic-clonic seizures. The patient was administered 1 g of levetiracetam and an injection of diazepam, after which the episode subsided. However, before she could recover from the postictal phase, she experienced another episode of GTCS. The patient was given additional levetiracetam and diazepam, and she stabilized after this.

Upon taking a detailed history, the patient's relative reported that the patient has had a known seizure disorder since the age of four and has been on sodium valproate for treatment. However, she has never been evaluated for the underlying cause.

On examination, the patient was in the postictal phase and afebrile, with a blood pressure of 120/70 mmHg, a heart rate of 120 beats per minute, and no focal neurological deficits.

To evaluate the cause of the seizure, all routine blood investigations were done, which were within normal limits (Table 1).

**Table 1:** Depicts the lab reports of the patient with the reference value.

Laboratory investigation	Value	Biological reference of lab
Hemoglobin	12.5	13-15 g/dL
Total leucocyte count	5,600	4,000-11,000/mm^3^
Platelet	245,000	150,000-450,000/mm^3^
Mean corpuscular volume	87.5	79-100 fL
Hematocrit	31.8	35%-40%
Urea	12	9-20 mg/dL
Creatinine	0.5	0.6-1.2 mg/dL
Sodium	133	137-144 mmoL/L
Potassium	4.2	3.5-5.0 mmoL/L
Calcium	8.4	8.4-10.2 mg/dL
Magnesium	2.1	1.6-2.3 mg/dL
Chloride	100	98-107 mmoL/L
Phosphorus	3.7	2.5-4.5 mg/dL
Serum ionic calcium	4.0	4.4-5.6 mg/dL
Uric acid	3.2	2.5-6.5 mg/dL
Thyroid-stimulating hormone	1.68	0.465-4.68 micro IU/mL
Homocysteine	8.62	4.7-12.6 micromol/L

**Figure 1:**
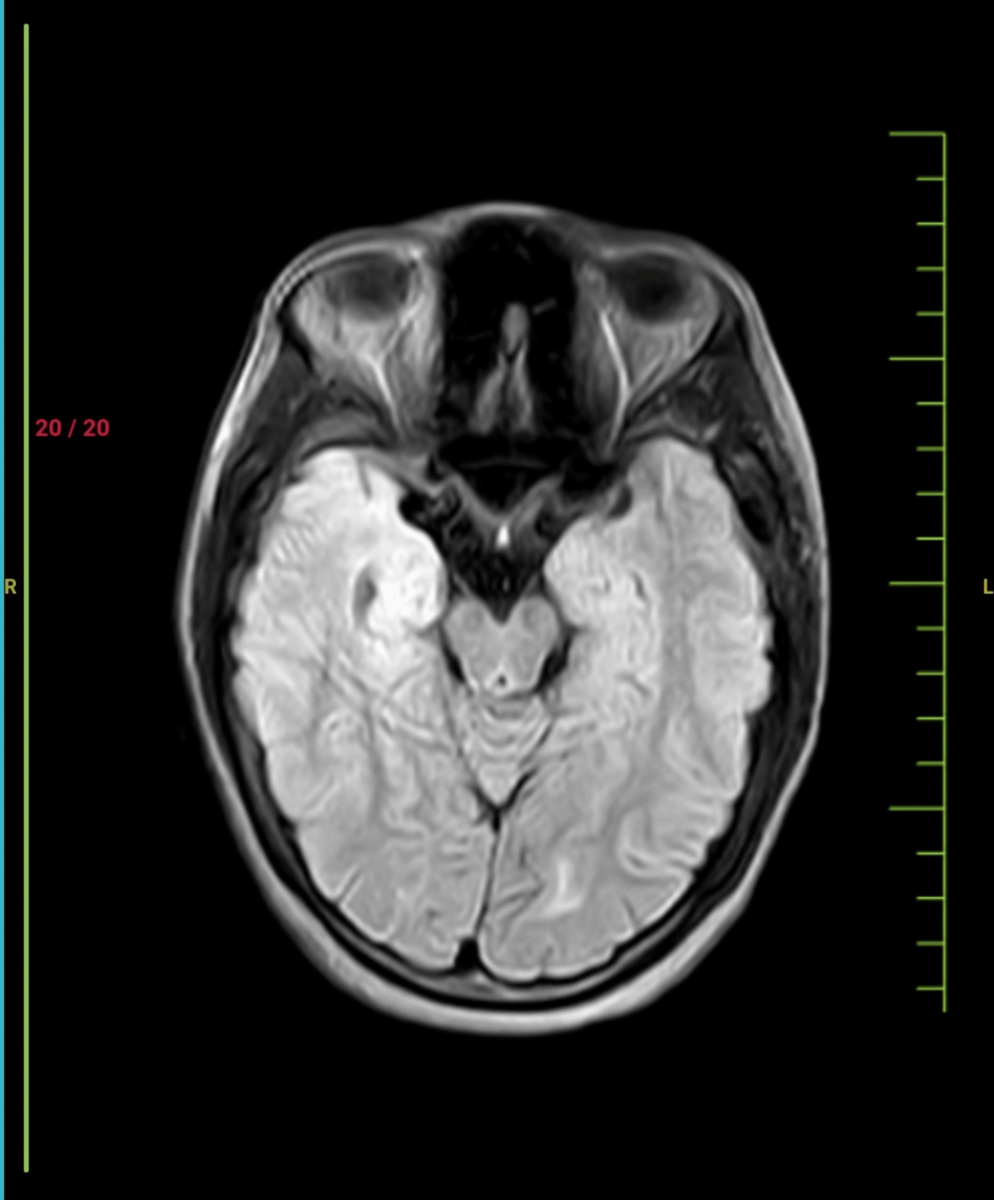
Axial fluid-attenuated inversion recovery sequence of MRI brain of the patient showing no obvious abnormality in the brain parenchyma.

**Figure 2:**
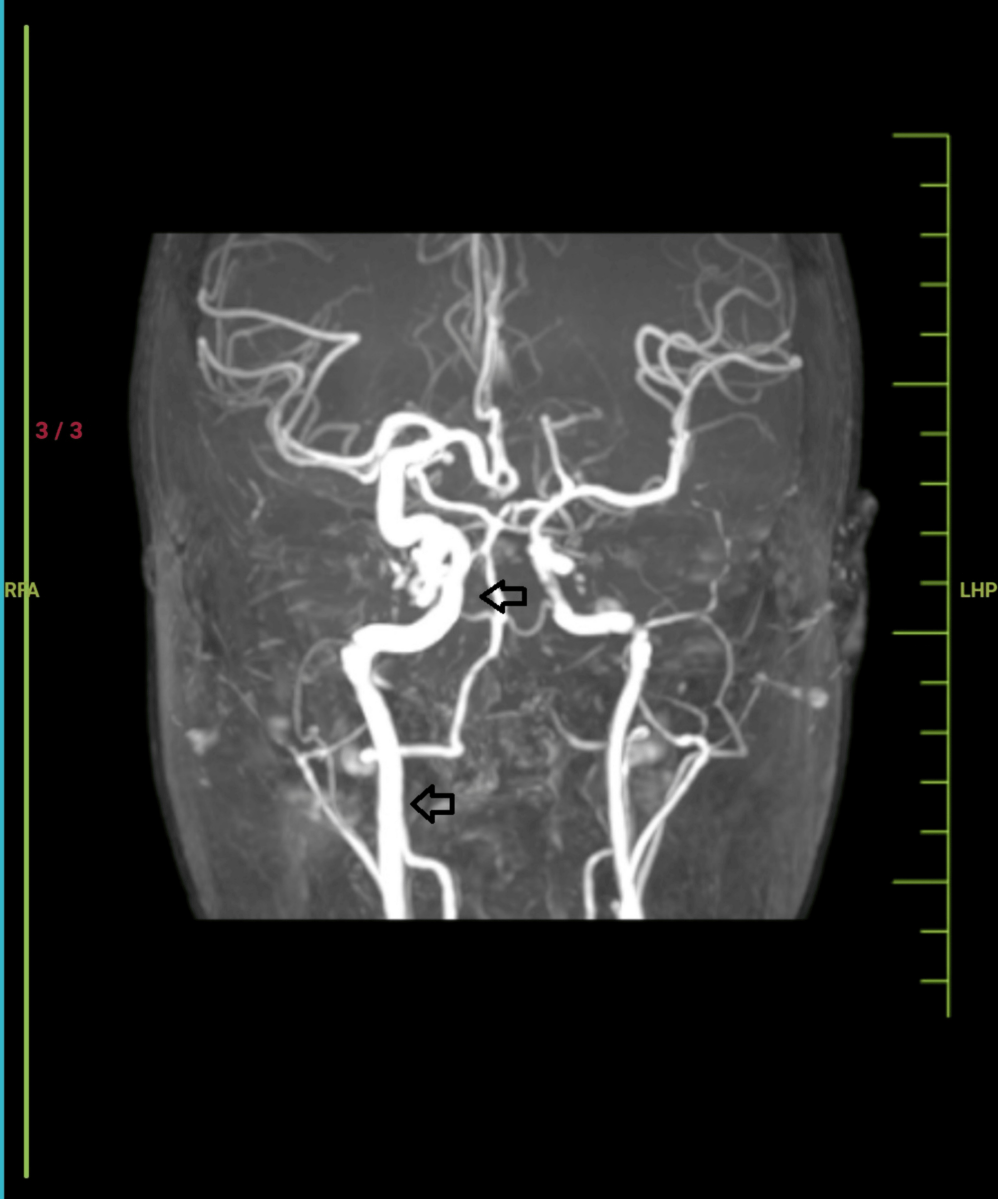
The coronal section of the MRI brain with MR angiography showing the dilated M1 segment of the right internal carotid artery (black arrows).

**Figure 3:**
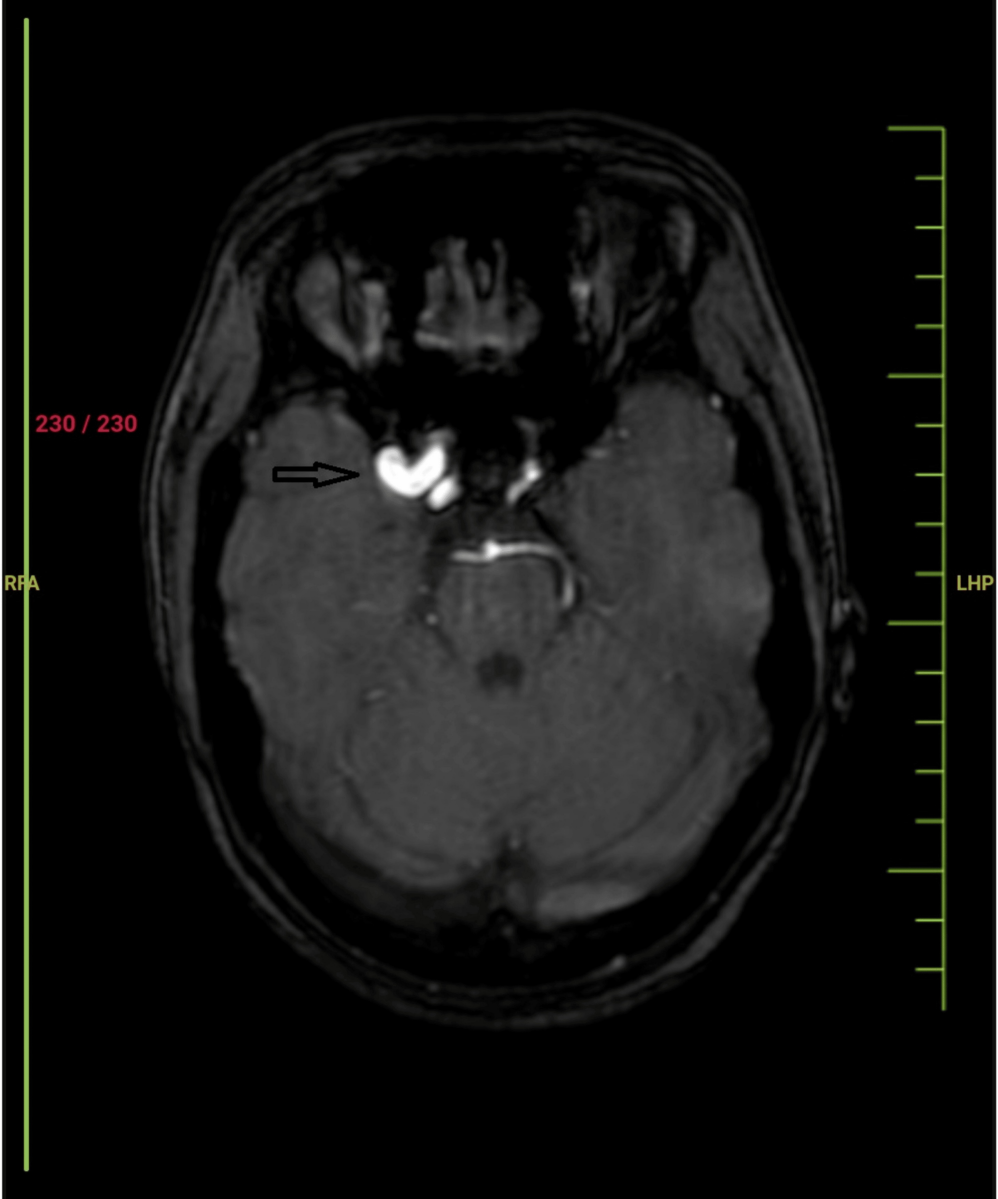
The MR angiography of the patient showing a dilated middle cerebral artery on the right side (black arrow).

For further evaluation of the cause of the seizures, the patient underwent an MRI of the brain with MR angiography, which suggested ectasia of the right internal carotid artery (ICA) and the M1 segment of the right middle cerebral artery (MCA). The maximum caliber of the right MCA was 6.5 mm compared to 3.8 mm on the left side, and the ICA measured 7.2 mm on the right side versus 5.7 mm on the left side, with no other abnormalities in the brain parenchyma (Figures 1-3).

To complete the evaluation of the seizure, an electroencephalogram was done, which showed the presence of generalized spikes and waves.

The patient was treated with intravenous levetiracetam 1 gm IV twice daily and lacosamide 200 mg IV twice daily for three days. After completing the intravenous treatment, the patient was discharged with oral forms of the same medications, along with a single antiplatelet (aspirin). The patient was followed up after three months and reported no new episodes of seizures during this period.

## Discussion

The term *cranial arterial dolichoectasia* derives from the Greek words *ektasis*, meaning dilation, and *dolikhós*, meaning elongation, referring to the dilatation and elongation of the cerebral arteries. There is a range of intracranial dilatation, from typical fluctuations to major aneurysmal dilatation that can result in life-threatening consequences [4]. DE and fusiform aneurysms are similar in that they are both nonsaccular aneurysms causing circumferential ballooning of a vascular segment.

The terms DE and fusiform aneurysm are used interchangeably to refer to vascular dilatation and increased tortuosity; nevertheless, DE is typically used to denote a less prominent form of arterial dilatation. In contrast to DE, which involves less extreme widening with elongation, fusiform aneurysms involve extreme circumferential ballooning of the entire artery wall for a brief segment. The same patient was diagnosed with both fusiform aneurysm and DE, which were identified as distinct conditions in a paper by Baran et al. [5]. The extent and definition of DE are not entirely clear, leading to some confusion. Consequently, the term *dilatative arteriopathy* has gained popularity in recent research on DE, emphasizing dilatation as the primary pathogenic feature.

The diagnostic criteria for DE about the anterior circulation are less defined. In a case of DE involving the circle of Willis reported by Baran et al. [5,6], the internal carotid artery (ICA) measured 11 mm and the MCA measured 7 mm. However, no specific guidelines were provided. An instance of MCA DE measuring 12.5 mm was reported by Fielies and Walker [7,8]. However, they did not specify any criteria. In these cases, the significant dilation of the arteries made visual examination simpler and more precise. However, for circumstances that are in the middle, there are not many precise standards. This makes sense because there are more studies on posterior circulation DE than anterior circulation DE, which is less common. Passero and Rossi suggested diameter cutoffs indicate *ectasia* as follows: ICA ≥ 7 mm, MCA ≥ 4 mm, and vertebral artery ≥ 4 mm [9].

Using the above criteria, the patient had ICA and MCA DE, with the MCA measuring 6.5 mm and the ICA measuring 7.2 mm.

The therapy for intracranial DE involves several considerations [10,11]. In individuals with DE, risk factors for ischemic stroke include arterial hypertension, male sex, advanced age (over 60), and a history of myocardial infarction. Currently, there are no established protocols for treating intracranial DE. While antiplatelet or anticoagulant medications are frequently recommended for DE patients with cerebral ischemia, there is little discussion about managing related risk factors or preventing potential bleeding.

## Conclusions

This case highlights the importance of considering DE as a differential diagnosis for seizures in young children. As in this case, had the patient been evaluated for seizures at a younger age, the morbidity caused by the seizures could have been greatly reduced. Diagnosing DE as soon as possible and performing the necessary imaging studies to rule out future complications is imperative. This case also highlights the necessity of a multidisciplinary approach to ensure comprehensive care and better outcomes for individuals with complex connections between neurological and vascular disorders. This case also sheds light on the rare but possible occurrence of anterior circulation DE. Further research and clinical awareness are necessary to better understand the pathophysiological pathways linking DE to seizures or anterior circulation DE to seizures and develop focused therapeutic options.
